# Development of an Affordable ELISA Targeting the SARS-CoV-2 Nucleocapsid and Its Application to Samples from the Ongoing COVID-19 Epidemic in Ghana

**DOI:** 10.1007/s40291-023-00655-0

**Published:** 2023-07-18

**Authors:** Kesego Tapela, Precious C. Opurum, Franklin Y. Nuokpem, Becky Tetteh, Godfred K. Siaw, Maria V. Humbert, Sylvia Tawiah-Eshun, Anna Ibrahim Barakisu, Kwame Asiedu, Samuel Kojo Arhin, Aaron A. Manu, Sekyibea N. A. Appiedu-Addo, Louisa Obbeng, Darius Quansah, Sylvester Languon, Claudia Anyigba, Daniel Dosoo, Nelson K. O. Edu, Daniel Oduro-Mensah, William Ampofo, Emmanuel Tagoe, Osbourne Quaye, Irene Owusu Donkor, Jewelna Akorli, Yaw Aniweh, Myron Christodoulides, Joe Mutungi, Yaw Bediako, Julian C Rayner, Gordon A Awandare, Christopher J. McCormick, Peter Kojo Quashie

**Affiliations:** 1 West African Centre for Cell Biology of Infectious Pathogens, College of Basic and Applied Sciences, University of Ghana, Legon, Accra, Ghana; 2 Department of Biochemistry, Cell and Molecular Biology, University of Ghana, Legon, Accra, Ghana; 3 The Francis Crick Institute, 1 Midland Rd, London, NW1 1AT UK; 4 School of Clinical and Experimental Sciences, Faculty of Medicine, University of Southampton, Southampton, SO16 6YD UK; 5 Yemaachi Biotech Inc., 222 Swaniker St, Accra, Ghana; 6 Noguchi Memorial Institute for Medical Research, University of Ghana, Legon, Accra, Ghana; 7 Department of Medical Laboratory Sciences, School of Biomedical and Allied Health, College of Health Sciences, University of Ghana, Accra, Ghana; 8 School of Clinical and Experimental Sciences, Faculty of Medicine, University of Southampton, Southampton, SO16 6YD UK; 9 Cambridge Institute for Medical Research, Cambridge Biomedical Campus, University of Cambridge, Cambridge, UK

## Abstract

**Introduction:**

The true nature of the population spread of severe acute respiratory syndrome coronavirus 2 (SARS-CoV-2) in populations is often not fully known as most cases, particularly in Africa, are asymptomatic. Finding the true magnitude of SARS-CoV-2 spread is crucial to provide actionable data about the epidemiological progress of the disease for researchers and policymakers. This study developed and optimized an antibody enzyme-linked immunosorbent assay (ELISA) using recombinant nucleocapsid antigen expressed in-house using a simple bacterial expression system.

**Methods:**

Nucleocapsid protein from SARS-CoV-2 was expressed and purified from *Escherichia coli*. Plasma samples used for the assay development were obtained from Ghanaian SARS-CoV-2 seropositive individuals during the pandemic, while seronegative controls were plasma samples collected from blood donors before the coronavirus disease 2019 (COVID-19) pandemic. Another set of seronegative controls was collected during the COVID-19 pandemic. Antibody detection and levels within the samples were validated using commercial kits and Luminex. Analyses were performed using GraphPad Prism, and the sensitivity, specificity and background cut-off were calculated.

**Results and Discussion:**

This low-cost ELISA (£0.96/test) assay has a high prediction of 98.9%, and sensitivity and specificity of 97% and 99%, respectively. The assay was subsequently used to screen plasma from SARS-CoV-2 RT-PCR-positive Ghanaians. The assay showed no significant difference in nucleocapsid antibody levels between symptomatic and asymptomatic, with an increase of the levels over time. This is in line with our previous publication.

**Conclusion:**

This study developed a low-cost and transferable assay that enables highly sensitive and specific detection of human anti-SARS-CoV-2 IgG antibodies. This assay can be modified to include additional antigens and used for continuous monitoring of sero-exposure to SARS-CoV-2 in West Africa.

**Supplementary Information:**

The online version contains supplementary material available at 10.1007/s40291-023-00655-0.

## Key Points

**Table:** 

A highly specific, sensitive and cost-effective (£0.96/test) immunoassay for the detection of exposure to SARS-CoV-2 was developed.
This assay identifies individuals who have been infected with SARS-CoV-2 from those with no previous exposure.
The protocol is easy to run and can be easily applied in other developing countries to detect previous exposure to SARS-CoV-2 patients from Ghana.

## Introduction

Coronavirus disease 2019 (COVID-19) remains a public health concern as its aetiological agent, severe acute respiratory syndrome coronavirus 2 (SARS-CoV-2) continues to mutate and produce variants that evade immune responses generated from past infections and vaccination [1, 2]. The extent and nature of the spread of SARS-CoV-2 in populations is often not fully understood as a significant proportion of cases, particularly in Africa, are asymptomatic, and testing is limited in many low-to-middle-income countries [3–5]. Finding the true extent of SARS-CoV-2 spread is critical to provide actionable data about the epidemiological progress of the disease for researchers and policymakers [6].

Reverse-transcription polymerase chain reaction (RT-PCR) targeting the nucleic acid of SARS-CoV-2 in nasopharyngeal or oropharyngeal samples is the gold standard for SARS-CoV-2 detection [7, 8]. Other techniques such as lateral flow devices (LFDs) detecting specific SARS-CoV-2 antigens have also been extensively employed, and they are generally more affordable and scalable than RT-PCR [9]. However, all these techniques only detect current viral infections, so unless testing happens regularly regardless of symptoms, some infections, particularly those that are asymptomatic, may be missed due to the short incubation time of SARS-CoV-2 [10]. Other limitations of these techniques include the high cost of reagents, the need for sophisticated sample storage, trained personnel, and delays between sampling and obtaining results [7]. There is, therefore, a clear need for the development of easy, low-cost and fast assays to screen for population exposure, with high sensitivity and specificity, especially for African countries and other resource-limited settings.

Enzyme-linked immunosorbent assay (ELISA) is an in vitro diagnostic tool which has significant advantages due to its simplicity, utility, low cost, high sensitivity and specificity [11]. It is also a common tool for seroprevalence studies [12] where antibody levels are measured in blood plasma/serum samples essentially for the assessment of viral dissemination and immunity timeline in a community [11].

The SARS-CoV-2 virus has two important proteins – the spike and nucleocapsid, both of which have been used widely for immunoassays [13, 14]. However, basing ELISA assays on spike protein can be impacted by the high rate of variation of this protein within different virus families, which can limit antibody cross-reactivity [1]. Moreover, it has been demonstrated that anti-nucleocapsid antibodies tend to last longer than anti-spike antibodies [15]. The SARS-CoV-2 nucleocapsid may therefore be a better target for ELISA assays, as they are highly abundant, are immunogenic [16, 17] and provide a sensitive target for serology studies [15, 18]. Immunoglobulin G (IgG) antibodies against nucleocapsid are usually detectable after 8 days of infection, making it a good target for detection of viral exposure in seroprevalence studies [17, 19].

In this study, we developed and optimized an antibody ELISA using nucleocapsid antigen expressed in-house using  a simple bacterial expression system, making it a potentially transferrable approach for tracking SARS-CoV-2 spread in resource-limited countries. This ELISA was further used to test panels of plasma from COVID-19 seropositive and negative individuals. It offers a low-cost option for tracking SARS-CoV-2 spread in developing countries.

## Materials and Methods

### Study Area, Design and Participants’ Descriptions

#### Ethics Approval and Consent to Participate

The study protocol was reviewed and approved by the Ethics Board of the College of Basic and Applied Sciences, University of Ghana (ECBAS 063/19-20), and the Ethical Review Committee of the Ghana Health Service (GHS-ERC 011/03/20). Additional formal written permissions were obtained from all collection sites. Participation was voluntary, and written informed consent was obtained from all the participants prior to enrolment.

#### Study Design, Sample Collection and Preparation

Participants were recruited among the Ghanaian population during the ongoing COVID-19 pandemic. This study was a mixed-methods study consisting of a cross-sectional and longitudinal study. Samples were obtained from the Ga East Regional Hospital, The Greater Accra Regional Hospital in Accra, Cape Coast University Hospital, Tamale Teaching Hospital, West Gonja Hospital, Seventh Day Adventist Hospital Asamang, Methodist Hospital Wenchi, and St. Luke Catholic Hospital Apam. Individuals with active SARS-CoV-2 infection were identified on the basis of quantitative reverse-transcription PCR (RT-PCR) results, and blood samples were collected immediately after PCR confirmation of SARS-CoV-2 infection, or concurrently with sample acquisition for PCR, then included in the study once a positive result had been obtained.

Blood (5 mL) was collected into ethylenediaminetetraacetic acid (EDTA) tubes using the venipuncture method. Plasma was isolated from whole blood using centrifugation, as previously described, with minor modification [5]. Briefly, whole blood samples were centrifuged for 5 min at 1000*g*. Plasma from the interface was then collected and stored at –80 °C for subsequent analyses. The peripheral blood mononuclear cells (PBMCs) were further processed, suspended in a freezing medium (FBS with 10% DMSO) and stored for future use.

#### Samples Used in Assay Development

Plasma samples used as a positive control (*n* = 100) were prepared from blood collected from individuals from the Ghanaian population who had tested positive for SARS-CoV2 infection using RT-PCR in 2020–2021. Negative controls (*n* = 100) were plasma samples collected before the COVID-19 pandemic (2017–2018) from blood donors at Korle Bu teaching hospital (Accra, Ghana) blood bank, and from individuals (*n* = 42) who tested negative for SARS-CoV-2 infection using RT-PCR during the early wave in Ghana (March–May 2020). These negative individuals were tested every 3 days for 2 weeks to confirm their true negativity.

#### Samples Used in Assay Development

In total, 370 samples were screened, of which 295 were symptomatic and 75 were asymptomatic (Table S1). A subset of 53 (symptomatic *n* = 43 and asymptomatic *n* = 10) who consented to follow-up sampling were sampled for up to 1 month. Samples were obtained from the following hospitals in Ghana: The Greater Accra Regional Hospital in Accra, Cape Coast University Hospital, Tamale Teaching Hospital, West Gonja Hospital, Seventh Day Adventist Hospital Asamang, Methodist Hospital Wenchi, and St. Luke Catholic Hospital Apam. All participants were above the age of 4, not pregnant, and written informed consent was obtained when blood samples were collected.

Symptomatic participants were defined as those that tested positive for SARS-CoV-2 with COVID-19 symptoms, while asymptomatic cases were defined as individuals who tested positive for SARS-CoV-2 but had no reported COVID-19 symptoms. The patient’s disease status was taken from medical reports and through a questionnaire.

### Validation of Seropositive Samples by Luminex Multiplex Assay and Rapid Diagnostic Tests (RDTs)

#### SARS-CoV-2 Specific Antibody Quantification Using Luminex Multiplex Assay

The levels of antibodies against SARS-CoV-2 nucleocapsid proteins were quantified as previously described [20, 21]. SARS-COV-2 nucleocapsid protein was coupled to Luminex beads, following an established protocol (hal-01299922 [22] courtesy of National Research Institute for Sustainable Development, France, https://en.ird.fr/). A 25 µL solution containing coupled beads was aliquoted into each well of a 96-well plate. The supernatant was removed while beads were immobilized using a magnetic holder and 1/100 diluted blood plasma was then added to each well. The plate was incubated for 2 h at 25 °C on a plate shaker at 400 rpm, then washed three times with wash buffer. Anti-human lgG secondary antibody (4 µg/mL) was added to the plate and incubated for 30 min on a Corning^®^ LSE™ digital microplate shaker at 400 rpm. This was followed by three washes, the addition of 1 µg/mL streptavidin–phycoerythrin (Invitrogen) and incubation for 10 min on a plate shaker at 400 rpm. Finally, the plates were read on a Luminex MAGPIX system (Luminex Corporation, Austin, TX, USA) using xPONENT™ software (V.4.3.229).

#### SARS-CoV-2 Specific Antibody Quantification Using RDTs and Wantai ELISA Kit

The presence of SARS-CoV-2 specific antibodies was detected using the ‘UNSCIENCE COVID-19 IgG/IgM antibody Rapid Test Kit’ (catalogue number UNCOV-40, lot number 20200326) following the manufacturer’s protocol. This is a strip-in-cassette lateral flow rapid diagnostic test kit which simultaneously detects IgM and IgG antibodies against SARS-CoV-2 nucleocapsid. Briefly, about 10 µL of plasma samples were put into the cassette followed by three drops of the kit’s buffer and results were read after 15 min. This kit had a manufacturer-declared IgG sensitivity and specificity of over 98% (https://covid-19-diagnostics.jrc.ec.europa.eu/devices/detail/634).

For evaluation using WANTAI SARS-CoV-2 Ab ELISA (Beijing Wantai Biological Pharmacy Enterprise Co., Ltd, China), the samples were also processed following the manufacturer’s protocol. Briefly, 100 μL of plasma samples was added into the provided plates, incubated at 37 °C for 30 min and washed five times using the kit buffer. This was followed by the addition of 100 μL HRP-conjugate, incubation at 37 °C for 30 min, and five washes. A substrate of 50 μL was added in each well and incubated at 37 °C for 15 min, and the reaction was stopped with 50 μL of stop solution. The absorbance was then measured at 450 nm using a Varioskan ELISA plate reader.

### Expression and Purification of SARS-CoV-2 Nucleocapsid Protein

#### Nucleocapsid-SUMO-His-Tag Expression and Purification

SARS-CoV-2 nucleocapsid protein was expressed in, and purified from, *Escherichia coli* as previously reported [9, 23]. Briefly, the plasmid pHYRSF53 (addgene #64696) encoding the SARS-CoV-2 nucleocapsid gene (NCBI Gene ID: 43740575) fused upstream to the SUMO-His-tag gene was transformed into BL21 (DE3). Transformants were grown at 37 °C in lysogeny broth (Miller) with 50 mg/mL kanamycin (Corning™, MT61176RG) under a shaking condition of 225 rpm. Recombinant protein expression was induced with 1 mM isopropyl-β-d-thiogalactopyranoside (Cayman, 367-93-1) at culture OD_600nm_ of 0.6 for 4 h and harvested.

The harvested bacteria were lysed using a lysis buffer (20 mM HEPES, 50 mM NaCl, 10% v/v glycerol, 1 mM β-mercaptoethanol, 1× protease inhibitor, 0.1% v/v Triton-X-100) and sonication. The soluble fraction was harvested and treated with 10 mM imidazole, and the recombinant protein was bound to the Nickel-NTA beads (Ni SeptFast™) (180202-100 mL, BioToolomics), washed, and eluted with 300 mL imidazole (56750-1 KG, Sigma-Aldrich) at high salt (1 M NaCl) and low salt (0.5 M NaCl) conditions into 1 mM EDTA (EDS-500G, Sigma-Aldrich). The protein was concentrated using an Amicon ultra-15 30 kDa cut-off centrifugal filter unit (UFC910024, Sigma-Aldrich), and the concentration was determined using Pierce™ BCA Protein Assay Kit (23227, Thermo Scientific™). The protein was resolved by 12% SDS-PAGE.

#### Ubiquitin-Like Protease (ULP) Expression, Purification and Cleavage of SUMO His-Tag

A plasmid encoding Ubiquitin-like protease-1 (ULP-1) was transformed into BL21 (DE3) and expressed in-house as previously done [23]. Tag-free nucleocapsid was obtained by incubating the SUMO His-tag fused nucleocapsid with ULP-1. Ubiquitin-like protease-1 (ULP-1) was added to the purified nucleocapsid protein at a ratio of 2 µg of ULP-1 to 1 mg of nucleocapsid protein with an addition of β-mercaptoethanol (21985023, Thermo Scientific™) to a final concentration of 5 mM. The solution was incubated on a rotary shaker overnight at 4 °C. Uncleaved nucleocapsid protein was removed by Ni-NTA IMAC with cleaved nucleocapsid protein being captured in the flow-through. The cleaved nucleocapsid was concentrated and stored at –20 °C for immediate use or –80 °C for the long term.

### Development and Optimization of the Indirect ELISA

Flat-bottom microtitre plates (Nunc Maxisorp plates) were coated overnight at 4 °C with 50 µL per well of nucleocapsid protein diluted in phosphate-buffered saline (PBS) (1 µg/mL). The plates were washed six times with PBS (524650, Sigma-Aldrich) containing 0.05% Tween 20 (PBST), blocked in 3% bovine serum albumin (BSA) (SLCJ7629, Sigma-Aldrich) in PBST at 200 µL per well for 1 h at room temperature followed by another six washes with PBST. Samples were prepared at 1:1000 dilutions in 1% BSA in PBST and added at 50 µL volume per well. A standard curve was generated by a 1/200 dilution of Human mAb to SARS-CoV-2 nucleocapsid (Ab 2722852, Abcam) two-fold across seven pairs of wells. After 2 h of incubation at room temperature, the wells were washed six times with PBST. Goat pAB to human IgG (HRP) (Ab 6858, Abcam) at a dilution of 1/15,000 in PBST was added at 50 µL volume per well and incubated for 1 h at room temperature. Following six washes with PBST, 100 µL of 1 step^TM^ ultra-3,3′,5,5′-tetramethylbenzidine (TMB) (WE3286221, Thermo Scientific) was added for 2 min for colour development. The reaction was stopped by the addition of 100 µL of 0.2 N sulphuric acid. The plates were read using the Varioskan ELISA plate reader at 450 nm wavelength. Optical densities were converted to concentrations using GraphPad Prism software Inc Version 9 (GraphPad Software, San Diego, California, USA).

### Statistical Analyses

Analyses were done using GraphPad Prism Version 9 (GraphPad Software, San Diego, California USA). Background cut-off values were determined as the mean of negative controls + (3 × standard deviation of negatives). The sensitivity was calculated as (the number of true positive/the total number of true positive + false negative samples) × 100, and the specificity was calculated as (the number of true negative/the total number of true negatives + false positive samples) × 100. The receiver-operating characteristic (ROC) was calculated to evaluate the performance of the assay.

### Background Cut-Off, Sensitivity and Specificity Calculations of the ELISA

1. $${\text{Sensitivity}} = 100 \times \frac{{{\text{np}}}}{{{\text{nTP}}}} ;\;100 \times \frac{97}{{100}} = 97\% .$$
2. $${\text{Specificity}} = 100 \times \frac{{{\text{nN}} - {\text{nFP}}}}{N} ;\;100 \times \frac{142 - 1}{{142}} = 99\% .$$
  where nP represents number of true positives detected by the ELISA, nTP represents total number of true positives, nN represents number of true negatives and nFP represents number of false positives detected by the ELISA

3. $${\text{Predictive}} \;{\text{value}} \;{\text{for}} \;{\text{negatives}} = \frac{{{\text{TP}}}}{{{\text{TP}} + {\text{FP}}}} \times 100;\;\frac{142}{{142 + 0}} \times 100 = 100\% .$$
4. $${\text{Predictive}}\;{\text{value}}\;{\text{for}}\;{\text{positives}} = \frac{{{\text{TN}}}}{{{\text{TN}} + {\text{FN}}}} \times \user2{ }100; \;\frac{100}{{100 + 1}} \times 100 = 99\% .$$

where TP represents true positive, FP represents false positive, TN represents true negative and FN represents false negative

5. Cut-off calculation
  $$\begin{gathered} {\text{Mean}}\; {\text{of}}\;{\text{negative }}\;{\text{controls}} + \left( {3 \times {\text{standard}}\;{\text{deviation}}} \right),\;{\text{where}}\;{\text{Mean}} = 10.02\;{\text{ and}}\;{\text{std.Deviation}} = 5.969 \hfill \\ ;10.02 + \left( {3 \times 5.969} \right) = 27.927\;\mu {\text{g/ml}}{.} \hfill \\ \end{gathered}$$

## Results

### Nucleocapsid Protein Purification

**Fig. 1:**
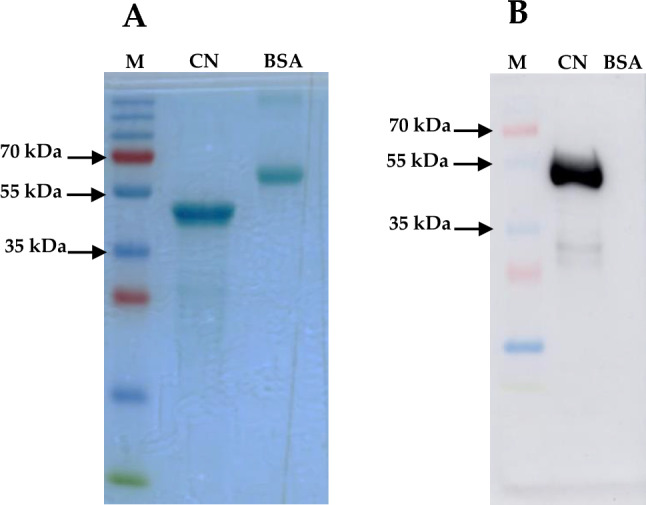
Purification of nucleocapsid protein from remnant SUMO-tag post ULP-1 cleavage. **A** A Coomassie-stained gel visualizing the nucleocapsid protein after 30 kDa filter unit-based purification (purity > 70%). **B** A western blot image that shows the recognition of the expressed, cleaved and purified SARS-CoV-2 nucleocapsid protein by a human anti-SARS-CoV-2 nucleocapsid mAb (ab273168, Abcam). *M *marker (PagerRuler^TM^ Plus), *CN *cleaved and purified nucleocapsid, *BSA *bovine serum albumin

Untagged nucleocapsid protein was purified to over 95% purity. The expression of the SUMO-His-tagged nucleocapsid protein was confirmed by its molecular weight following SDS-PAGE and western blotting analysis (Fig. S1). After cleavage of the SUMO-His tag (Fig. S2), the cleaved nucleocapsid protein was repurified and confirmed using an NC-targeted western blot (Fig. 1).

### Optimization of ELISA Protocol

**Fig. 2:**
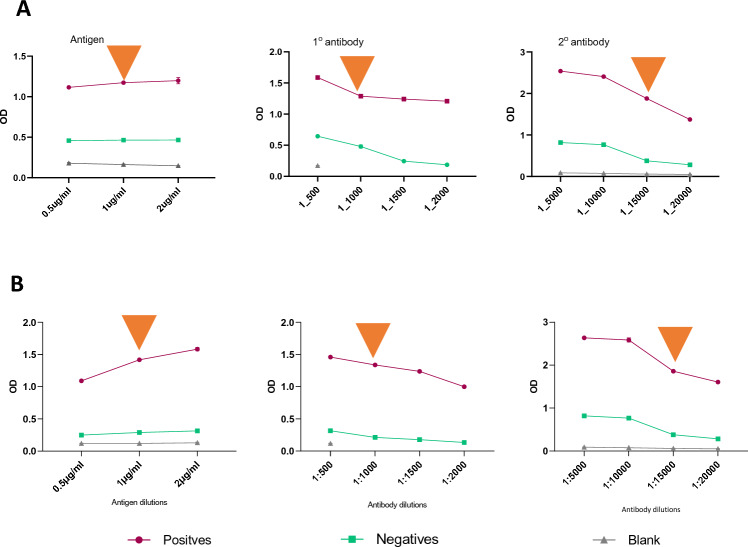
Optimization of the coating antigen, human mAb to SARS-CoV-2 nucleocapsid, and goat pAB to human IgG (HRP) using **A** commercial primary antibody and **B** pooled plasma. The OD450 of each concentration is shown by the line graph with the 25th and 75th percentiles. The orange arrows indicate the chosen suitable dilutions during optimizations

To develop the ELISA assay, seronegative (*n* = 142) and seropositive (*n* = 100) plasma samples were used as outlined in the Methods. The negative controls included both samples collected pre-pandemic (*n* = 100), and samples collected during the pandemic that tested negative by RT-PCR, Luminex and RDT assays (*n* = 42). To optimize the assay, it was important to provide sufficient antigen to capture antibodies, and for sufficient antibodies to be captured to avoid non-specific backgrounds. This study optimized the coating antigen, primary and secondary concentrations for the ELISA. The plates were initially coated with dose-ranging concentrations of SARS-CoV-2 full-length nucleocapsid protein (from 0.5 to 2 µg/mL), pooled plasma samples/human mAb to SARS-CoV-2 nucleocapsid (Ab 2722852, Abcam) (from 1/500 to 1/2000), and goat pAB to human IgG (HRP) (Ab 6858, Abcam) was used at different dilutions (1/5000 to 1/20,000) (Fig. 2). The fixed concentrations were as follows: for coating antigen optimization, primary and secondary antibody dilutions were 1/1000 and 1/15,000, respectively. For primary antibody optimization, antigen and secondary dilutions were 1 µg/mL and 1/15,000 respectively. When optimizing the secondary antibody, 1 µg/mL of antigen and 1/1000 of the primary antibody was used (Fig. 2). The pooled plasma samples were from ten seropositive samples with the highest antibody levels determined by Luminex multiplex assay and the Wantai ELISA kit (Table S3).

**Fig. 3:**
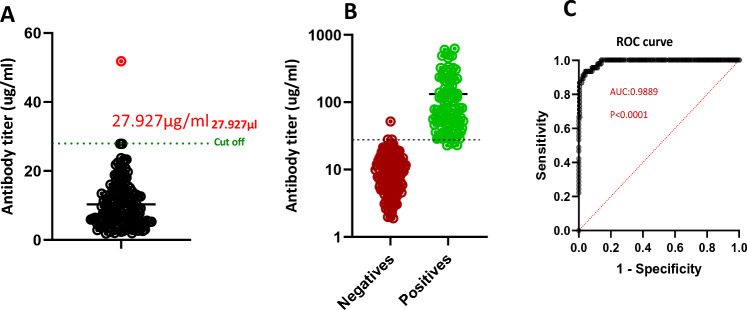
Establishing cut-off values and evaluating the ELISA. **A** A scatter plot showing the distribution of the negative samples (*n* = 142) relative to the cut-off value (mean + 3 × standard deviation), shown as a dashed line. **B** A scatter plot showing the distribution of both negative (*n* = 142) and positive (*n* = 100) samples along the cut-off value represented by a dashed line. **C** The ROC curve of the ELISA at a 95% confidence interval of 0.9781–0.9997

The OD_450_ was interpolated in a standard curve using Graph Prism v.9 to determine the antibody titre of the samples for both negative (*n* = 142) and positive (*n* = 100) samples. The titres were used to determine the cut-off value, which was calculated as mean + (3 × standard deviation) of all negative control samples and amounted to 27.927 µg/mL (Fig. 3a). The antibody titre of all negative samples apart from one (highlighted in red) fell below the cut-off value. This discrepant sample was one of the PCR-confirmed negatives (multiple timepoints) collected during the pandemic with no antibody detection by RDT. Only 3 of the 100 RT-PCR positive samples yielded titres that fell below the cut-off value (Fig. 3b). These samples tested positive for IgM, implying that IgG production had not as yet peaked.

Receiver operating characteristic (ROC) analysis was performed on the basis of the antibody titre, and the data revealed that there is a 98.9% chance the ELISA can distinguish between negative and positive samples, with sensitivity and specificity of 97% and 99%, respectively.

### Symptomatic Patients with COVID-19 Produce Anti-nucleocapsid Comparable to Asymptomatic Patients

**Fig. 4:**
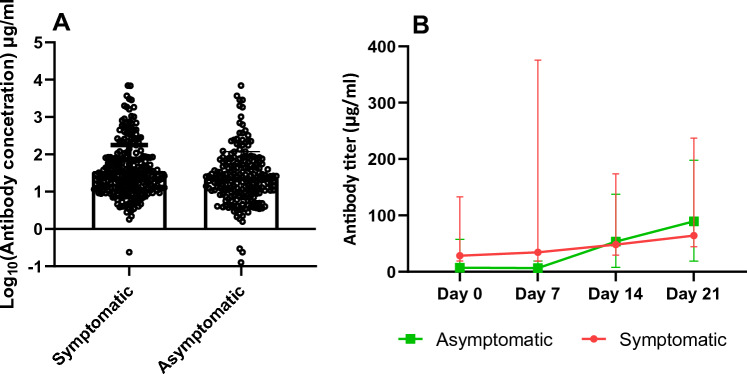
The nucleocapsid antibody concentration levels of patients with COVID-19. **A** Scatter plots with a bar showing comparisons of antibody concentration levels between symptomatic (*n* = 295) and asymptomatic patients (*n* = 75) individuals. **B** Change in antibody concentration levels over time. The antibody concentration levels were analysed from symptomatic (*n* = 43) and asymptomatic (*n* = 10) patients at different timepoints for 4 weeks. The median quantity of the antibody levels for each sampling day is shown by the line graph and the 25th and 75th percentiles are antibody levels. Statistical significance between groups was determined by a two-tailed Mann-Whitney *U* test (ns: *p* > 0.05, *p < 0.05, **p < 0.01, ****p < 0.0001)

The ELISA was used to quantify antibody concentrations in plasma from patients with COVID-19. The study demonstrated no differences in nucleocapsid antibody levels between symptomatic and asymptomatic  patients (Fig. 4A). The levels of antibody concentrations increased over time for both symptomatic and asymptomatic individuals (Fig. 4B).

## Discussion

COVID-19 caused by infection with SARS-CoV-2 is a worldwide challenge that continues to pose a threat to human beings. Seroprevalence studies are required to understand the real impact of the virus as most cases are asymptomatic and those with mild symptoms are mostly undetected [24]. The studies can also be used to detect previous infections, most importantly for unvaccinated people such as underage children or people refusing vaccinations. This can be important to validate an expected herd immunity [15]. Furthermore, seroprevalence studies help predict the level of protection against reinfection [25, 26]. Serology-based techniques such as ELISA have been useful for large-scale screening as they are advantageous over other techniques. Moreover, most of the expensive commercial kits available are qualitative rather than quantitative, and specificity and sensitivity remain uncertain as they usually require proper validation before use [27, 28]. Therefore, there is a need for reliable serology testing especially in developing countries such as Ghana. Our study purified a previously expressed SARS-CoV-2 full-length nucleocapsid protein and used it to develop and optimize an in-house ELISA protocol. The protocol detected and quantified nucleocapsid-specific human IgG in Ghanaian patients with COVID-19.

Our developed ELISA demonstrates 99% specificity and 97% sensitivity with an accuracy area under the curve (AUC) of 0.9896. This is in line with other in-house ELISA developed elsewhere [15]. However, when compared with readily available commercial kits, reports indicate that many have lower sensitivity and specificity [4, 29]. A study on SARS-CoV-2 positive individuals showed lower sensitivities of some commercial kits, e.g. 53.7% for Abbott, 93.1% for Roche, 77.1% for Euroimmun and 89.2% for Immundiagnostic [29]. In another study, a nucleocapsid-based kit (UNSCIENCE) exhibited a specificity of 94% [4] (Tables S3 and S4). Compared with these tests, this nucleocapsid-based ELISA provides high sensitivity (97%) and specificity (99%) and can be a suitable assay for seroprevalence studies. Moreover, nucleocapsid-based antigens are more suitable for immune assays as spike-based assays have been found to have a reduced sensitivity as compared with nucleocapsid assay [30]. Additionally, our ELISA is not only qualitative but also quantitative as compared with most commercial kits.

The other advantage of our ELISA is that it can potentially be transferable to other resource-limited countries. The assay protocol is simple with basic readily available reagents. It does not require complicated facilities and equipment to use; the only equipment needed to run the ELISA is the plate reader, which is easily accessible in most facilities. The samples used for validation of this ELISA are from an African population which can be easily applied for seroprevalence studies in other African countries. We, therefore, recommend that the assay can be transferred to other African countries.

The other key element of this ELISA is that it is of low cost. We have shared a cost calculation indicating that the approach of the assay is cheaper (0.95£/sample) (Fig. S2) to use as compared with most commercial kits which cost approximately 4.8–11.90 £/sample excluding shipping (https://www.abcam.com; https://www.thermofisher.com/). This ELISA, produced in Africa, will be a cheaper assay to use in resource-limited African countries.

We further utilized this ELISA to quantify the antibodies in Ghanaian patients with COVID-19, to compare the anti-nucleocapsid antibody levels between symptomatic and asymptomatic patients. Our study did not find any significant differences between symptomatic and asymptomatic patients. The antibody concentration levels increased over time for both groups. Even though there is not much literature comparing symptomatic and asymptomatic patients, higher anti-nucleocapsid was detected in critically ill patients with COVID-19 as compared with non-critically ill [31]. In another study in the Netherlands [32], higher anti-nucleocapsid antibodies were detected in moderate or severe cases while undetectable in asymptomatic patients. Our results may be different from previous studies due to the differences in the COVID-19 immune response between Africans and communities from other nations, which may not be unexpected [5].

One of the limitations of the study is the inability to use post-pandemic negative controls. Although it would have been ideal to have more negative samples, the 42 samples used represent a good population set, since these had corresponding Luminex data. Moreover, the pre-COVID samples are believed to present a better true negative. The other limitation was the use of RT-PCR for testing the presence of the virus. Studies have reported RT-PCR to be associated with risks of false negatives due to low viral load from patients in the early or late stages of infection [33, 34]. However, this was rectified by testing the negative individuals every 3 days for 2 weeks to confirm if they are true negatives.

## Conclusions

The ELISA protocol developed in this study is of low cost, is transferable and enables highly sensitive and specific detection of human anti-SARS-CoV-2 IgG antibodies. Having a valid in-house ELISA for COVID-19 in developing countries such as Ghana is good for population screening purposes. The nucleocapsid antibody responses of Ghanaian patients with COVID-19 appear unchanged regarding disease severity.

## Supplementary Information

Below is the link to the electronic supplementary material.

Supplementary file1 (DOCX 5356 KB)

## Declarations

### Data availability statement

The datasets used and/or analysed during the current study are available from the corresponding author upon reasonable request.

## References

[1] WHO. Tracking SARS-CoV-2 variants. World Health Organisation. 2022;:1–20. https://www.who.int/en/activities/tracking-SARS-CoV-2-variants/. Accessed 23 Nov 2022.

[2] Morang CM, Ngoi JM, Gyam J, Amuzu DSY, Nuertey BD, Soglo PM (2022). in Ghana from 2020–2021. Nat Commun.

[3] Osei SA, Biney RP, Anning AS, Nortey LN, Ghartey-Kwansah G (2022). Low incidence of COVID-19 case severity and mortality in Africa; could malaria co-infection provide the missing link?. BMC Infect Dis.

[4] Quashie PK, Mutungi JK, Dzabeng F, Oduro-Mensah D, Opurum PC, Tapela K (2021). Trends of severe acute respiratory syndrome coronavirus 2 (SARS-CoV-2) antibody prevalence in selected regions across Ghana. Wellcome Open Res..

[5] Tapela K, Oyawoye FO, Olwal CO, Opurum PC, Amponsah JA, Segbedzi KAL (2022). Probing SARS-CoV-2-positive plasma to identify potential factors correlating with mild COVID-19 in Ghana, West Africa. BMC Med.

[6] McConnell D, Hickey C, Bargary N, Trela-Larsen L, Walsh C, Barry M (2021). Understanding the challenges and uncertainties of seroprevalence studies for sars-cov-2. Int J Environ Res Public Health.

[7] Kevadiya BD, Machhi J, Herskovitz J, Oleynikov MD, Blomberg WR, Bajwa N (2021). Diagnostics for SARS-CoV-2 infections. Nat Mater.

[8] Yu F, Xie G, Zheng S, Han D, Bao J, Zhang D (2021). Assessment of the diagnostic ability of four detection methods using three sample types of COVID-19 patients. Front Cell Infect Microbiol.

[9] Victoria M, Chinonyerem P, Brendish NJ, Poole S, He P, Katis I (2022). A SARS-CoV-2 nucleocapsid ELISA represents a low-cost alternative to lateral flow testing for a community screening in LMI countries. J Infect.

[10] Marchi S, Viviani S, Remarque EJ, Ruello A, Bombardieri E, Bollati V (2021). Characterization of antibody response in asymptomatic and symptomatic SARS-CoV-2 infection. medRxiv..

[11] Peng P, Liu C, Li Z, Xue Z, Mao P, Hu J (2022). Emerging ELISA derived technologies for in vitro diagnostics. TrAC Trends Anal Chem.

[12] Klumpp-Thomas C, Kalish H, Drew M, Hunsberger S, Snead K, Fay MP (2021). Standardization of ELISA protocols for serosurveys of the SARS-CoV-2 pandemic using clinical and at-home blood sampling. Nat Commun.

[13] Grzelak L, Temmam S, Planchais C, Demeret C, Tondeur L, Huon C (2020). A comparison of four serological assays for detecting anti–SARS-CoV-2 antibodies in human serum samples from different populations. Sci Transl Med..

[14] Tehrani ZR, Saadat S, Saleh E, Ouyang X, Constantine N, DeVico AL (2020). Performance of nucleocapsid and spike based SARS-CoV-2 serologic assays. PLoS ONE.

[15] Luo J, Brakel A, Krizsan A, Ludwig T, Mötzing M, Volke D (2022). Sensitive and specific serological ELISA for the detection of SARS-CoV-2 infections. Virol J..

[16] Satarker S, Nampoothiri M (2020). Structural proteins in severe acute respiratory syndrome coronavirus-2. Arch Med Res.

[17] Liu PP, Zong Y, Jiang SP, Jiao YJ, Yu XJ (2021). Development of a nucleocapsid protein-based ELISA for detection of human IgM and IgG antibodies to SARS-CoV-2. ACS Omega.

[18] Leung DTM, Tam FCH, Chun HM, Chan PKS, Cheung JLK, Niu H (2004). Antibody response of patients with severe acute respiratory syndrome (SARS) targets the viral nucleocapsid. J Infect Dis.

[19] Horn MP, Jonsdottir HR, Brigger D, Damonti L, Suter-Riniker F, Endrich O (2022). Serological testing for SARS-CoV-2 antibodies in clinical practice: a comparative diagnostic accuracy study. Allergy Eur J Allergy Clin Immunol..

[20] Mensah BA, Ndong IC, Quashie PK, Guichet E, Abuaku B, Effah-Baafi Y (2022). Population-based seroepidemiological investigation of the dynamics of SARS-CoV-2 infections in the Greater Accra Region of Ghana. Sci Rep.

[21] Ayouba A, Thaurignac G, Morquin D, Tuaillon E, Raulino R, Reynes J (2020). Multiplex detection and dynamics of IgG antibodies to SARS-CoV2 and the highly pathogenic human coronaviruses SARS-CoV and MERS-CoV Ahidjo. J Clin Virol.

[22] Perraut R, Varela M, Mbengue B, Guillotte M, Perraut R, Varela M (2015). Standardization of a multiplex magnetic bead-based for simultaneous detection of IgG to plasmodium antigens to cite this version: HAL Id : hal-01299922 Journal of Immunological Techniques in Infectious Diseases Standardization of a Multiplex Magnetic Bea. J Immunol Tech Infect Dis..

[23] Guerrero F, Ciragan A, Iwaï H (2015). Tandem SUMO fusion vectors for improving soluble protein expression and purification. Protein Expr Purif.

[24] Gdoura M, Ben GF, Ben HM, Chamsa W, Triki H, Bahloul C. Development of an in-house quantitative ELISA for the evaluation of different Covid-19 vaccines in humans. Sci Rep. 2022;12:1–9. 10.1038/s41598-022-15378-1 PMC9252535 35788676

[25] Ramanathan K, Antognini D, Combes A, Paden M, Zakhary B, Ogino M (2021). Assessment of protection against reinfection with SARS-CoV-2 among 4 million PCR-tested individuals in Denmark in 2020: a population-level observational study. Lancet.

[26] Chen Q, Zhu K, Liu X, Zhuang C, Huang X, Huang Y (2022). The protection of naturally acquired antibodies against subsequent SARS-CoV-2 infection: a systematic review and meta-analysis. Emerg Microb Infect..

[27] Kohmer N, Westhaus S, Rühl C, Ciesek S, Rabenau HF (2020). Clinical performance of different SARS-CoV-2 IgG antibody tests. J Med Virol.

[28] Lisboa Bastos M, Tavaziva G, Abidi SK, Campbell JR, Haraoui LP, Johnston JC, et al. Diagnostic accuracy of serological tests for covid-19: Systematic review and meta-analysis. BMJ. 2020;370. 10.1136/bmj.m2516 PMC7327913 32611558

[29] Eberhardt KA, Dewald F, Heger E, Gieselmann L, Vanshylla K, Wirtz M (2021). Evaluation of a new spike (S)-protein-based commercial immunoassay for the detection of anti-SARS-CoV-2 IgG. Microorganisms..

[30] Barlev-Gross M, Weiss S, Ben-Shmuel A, Sittner A, Eden K, Mazuz N (2021). Spike vs nucleocapsid SARS-CoV-2 antigen detection: application in nasopharyngeal swab specimens. Anal Bioanal Chem.

[31] Brlić PK, Pavletić M, Lerga M, Krstanović F, Matešić MP, Miklić K (2022). SARS-CoV-2 spike and nucleocapsid antibody response in vaccinated Croatian healthcare workers and infected hospitalized patients: a single center cohort study. Viruses.

[32] Tutukina M, Kaznadzey A, Kireeva M, Mazo I (2021). IgG antibodies develop to spike but not to the nucleocapsid viral protein in many asymptomatic and light COVID-19 cases. Viruses.

[33] Lippi G, Simundic AM, Plebani M (2020). Potential preanalytical and analytical vulnerabilities in the laboratory diagnosis of coronavirus disease 2019 (COVID-19). Clin Chem Lab Med.

[34] Shihong Gao D, Zhu X, Lu B (2021). Development and application of sensitive, specific, and rapid CRISPR-Cas13-based diagnosis. J Med Virol.

