# Supplementary material for: Development of an Affordable ELISA Targeting the SARS-CoV-2 Nucleocapsid and Its Application to Samples from the Ongoing COVID-19 Epidemic in Ghana
Source: Mol Diagn Ther. 2023 Jul 18;27(5):583–92. doi: 10.1007/s40291-023-00655-0 (PMC10435612; doi:10.1007/s40291-023-00655-0)
Supplement: Supplementary file 1 — Supplementary file1 (DOCX 5356 KB) [file 40291_2023_655_MOESM1_ESM.docx]

**Supplementary Materials**


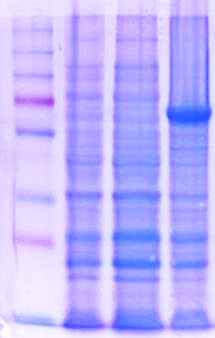


**250 kDa**

**130 kDa**

**100 kDa**

**70 kDa**

**55 kDa**

**35 kDa**

**25 kDa**

**M**

**Un**

**5µl**

**Un**

**10µl**

**In**

**5µl**

**A**


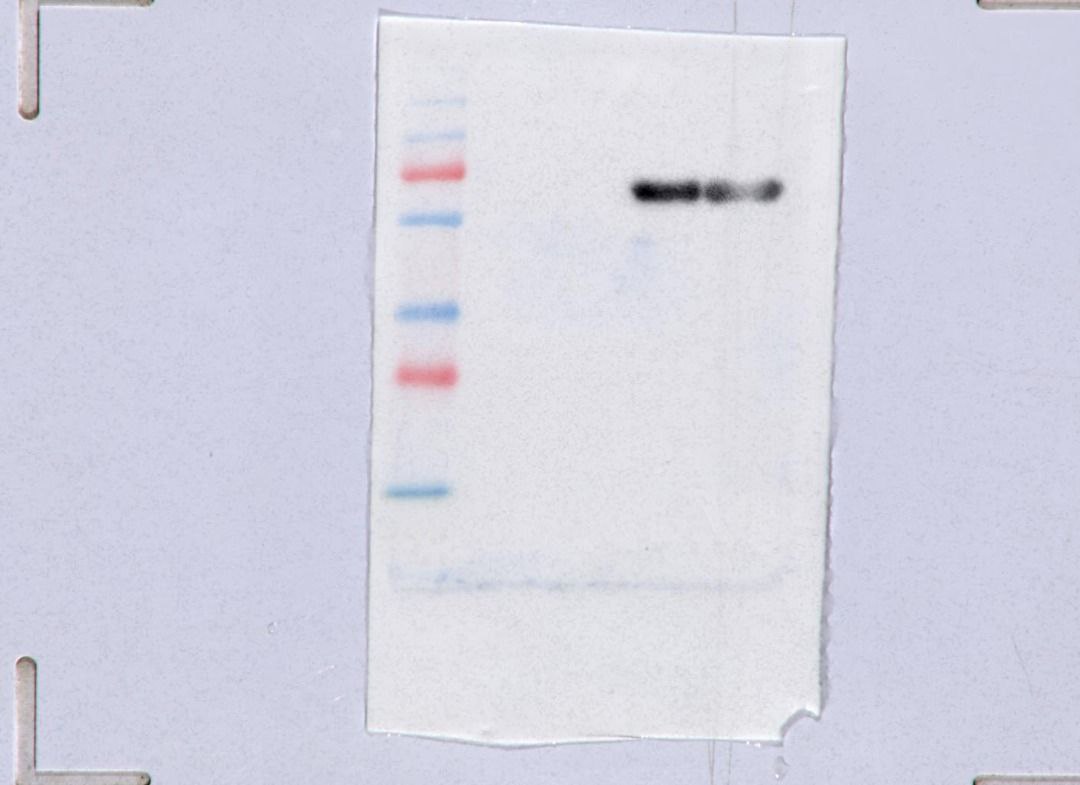


**70 kDa**

**55 kDa**

**M**

**Un**

**5µl**

**Un**

**10µl**

**In**

**5µl**

**B**


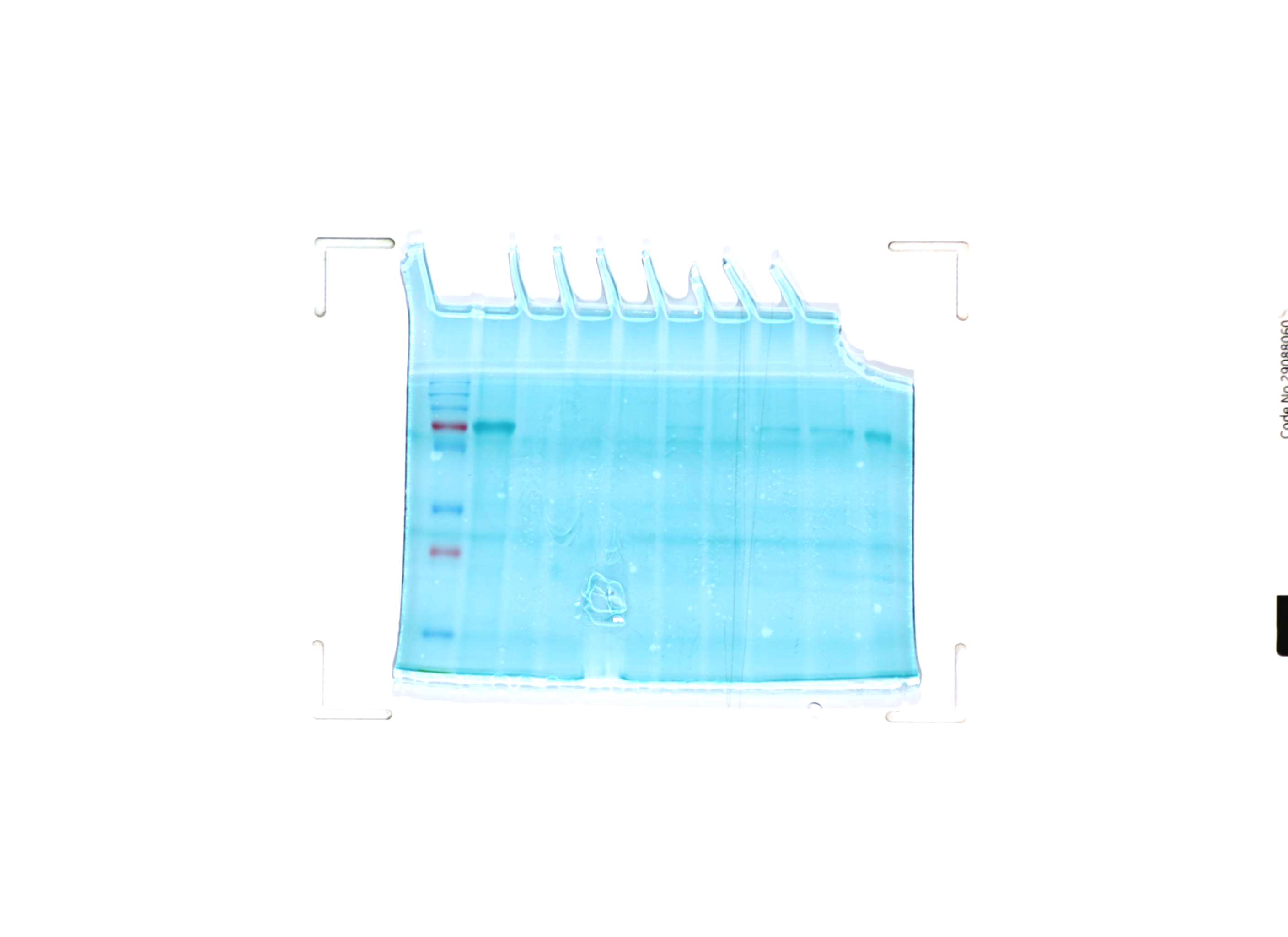


**70 kDa**

**55 kDa**

**M**

**Conc**

**C**

**M**=Marker (PageRuler^TM^ Plus); **Un**=Uninduced; **In**=Induced; **Conc**=Elution Concentrate

**Figure S1.** Expression of nucleocapsid protein fused to the SUMO tag: **A.** Shows a Coomassie-stained gel (SDS-PAGE) with uninduced and IPTG-induced sample lanes. The gel shows the expected overexpression in the induced lane compared to the uninduced lanes. The overexpressed product (highlighted in a red box) appears as a ~70 kDa protein; a size consistent with recombinant protein expected. **B.** Shows the recognition of the overexpressed band by a human anti-SARS-CoV-2 nucleocapsid mAb (ab273168, abcam) conjugated to HRP in a western blot. This confirms that the overexpressed protein is the nucleocapsid-SUMO-tagged protein. **C.** the purified and concentrated protein is resolved by SDS-PAGE and visualized by Coomassie staining.


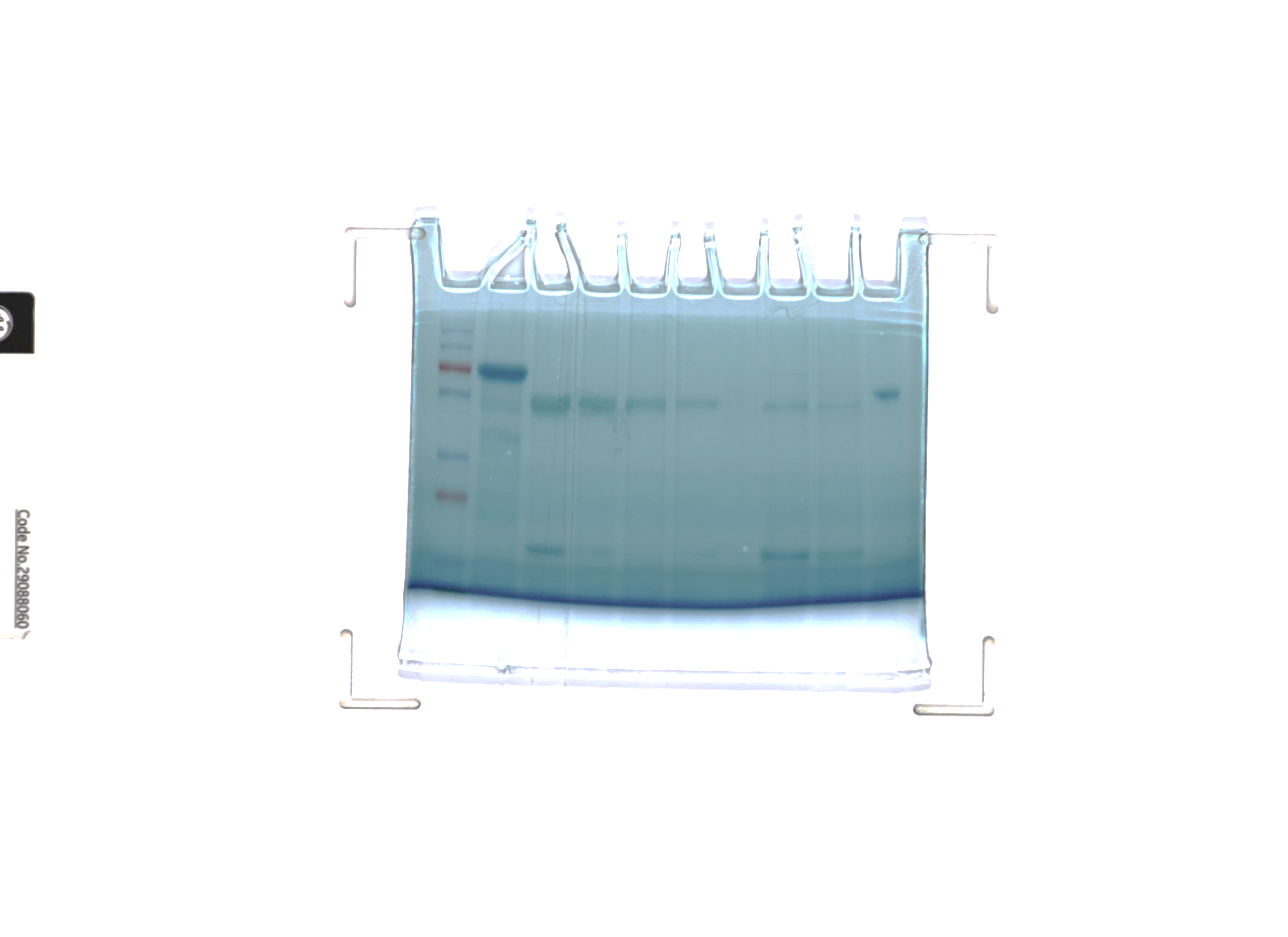


**100 kDa**

**70 kDa**

**55 kDa**

**35 kDa**

**25 kDa**

**15 kDa**

**M**

**UC**

**AC**

**FT**

**A**

**B**


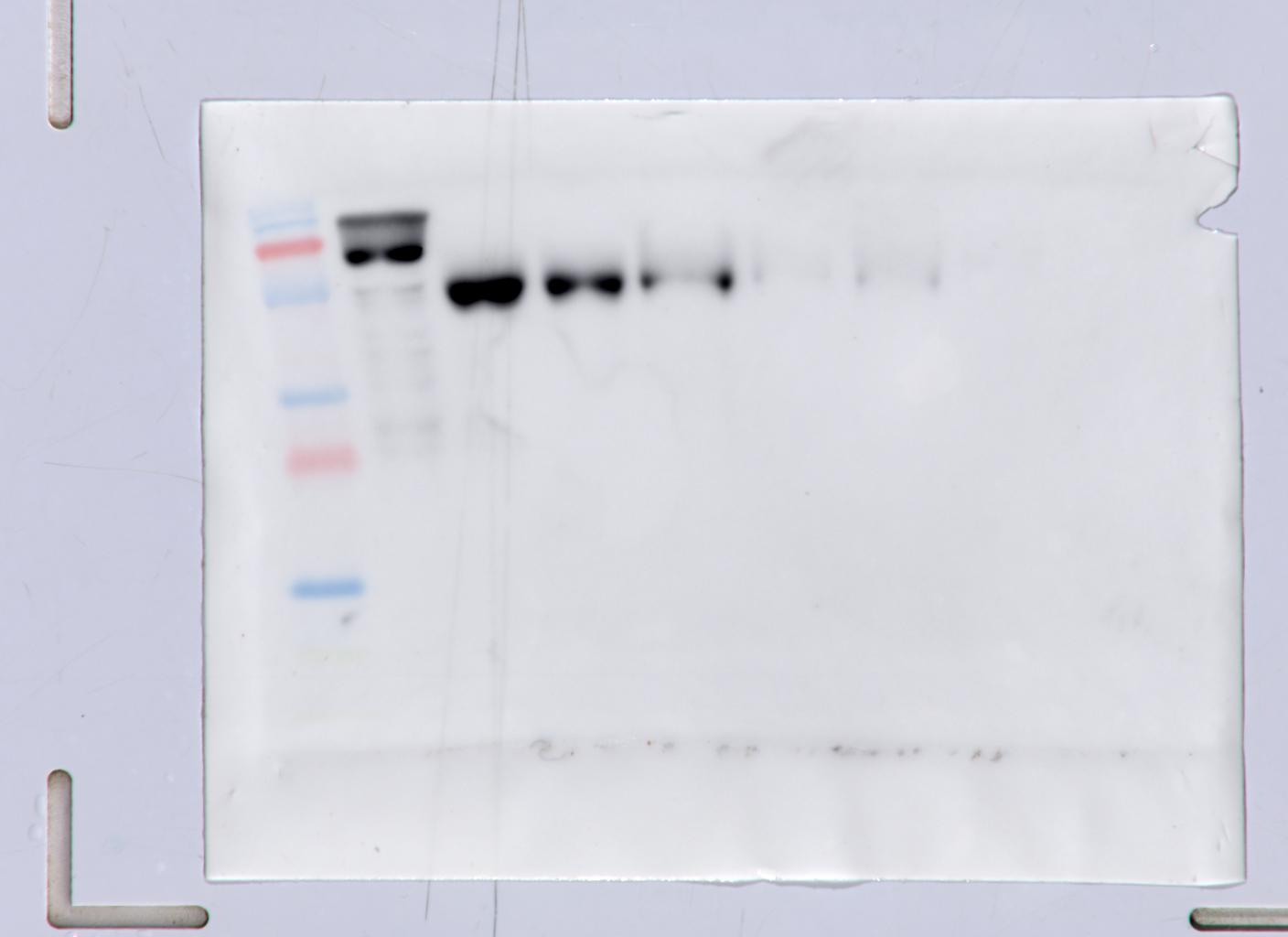


**70 kDa**

**55 kDa**

**M**=Marker (PagerRuler^TM^Plus); **UC**=Uncleaved (SUMO Nucleocapsid); **AC**=After Cleavage with ULP-1; **FT**=Flowthrough during purification post-cleavage

**Figure S2.** Cleavage of nucleocapsid-SUMO tagged protein by ULP-1 at the SUMO protease site: **A.** The Coomassie stained gel shows the uncleaved nucleocapsid protein (lane UC) and protein fragments post ULP1 (lane AC). The two fragments observed (highlighted in red boxes) correspond to the nucleocapsid (approximately 45–55 kDa) and SUMO tag (<28 kDa) fragments expected post-ULP-1 cleavage. **B**. The nucleocapsid fragment was confirmed by western blot (human anti_SARS-CoV-2 Nucleocapsid mAb).

|  | **Total samples (n=370)** |
| --- | --- |
| Median age, years (IQR)* | 38(13-89) |
| Male | 187(51%) |
| Female | 183(49%) |
| SpO_2_^º^ | 98 (94.5-98.75) |
| Clinical status |  |
| Asymptomatic | 75 |
| Symptomatic | 295 |

**Table S1. The characteristics of participants used for testing the ELISA.**

*When data was available ^º^symptomatic cases only

Table S2: Cost calculations of the ELISA

Table S3: Comparison of the ELISA with other assays

P= Positive; N=Negative

Table S4: Comparison of specificity and sensitivity of the ELISA with other assays
